# Bilateral Peritonsillar Abscesses: A Case Presentation and Review of the Current Literature with regard to the Controversies in Diagnosis and Treatment

**DOI:** 10.1155/2011/981924

**Published:** 2011-05-05

**Authors:** G. X. Papacharalampous, P. V. Vlastarakos, G. Kotsis, D. Davilis, L. Manolopoulos

**Affiliations:** ^1^ENT Department, Elpis General Hospital, 11522 Athens, Greece; ^2^ENT Department, Lister Hospital, 64 Morecambe Close, Stevenage, Hertfordshire SG1 2BF, UK; ^3^ENT Department, University of Athens, Hippokration General Hospital, 11527 Athens, Greece

## Abstract

Although unilateral peritonsillar abscess is a common complication of acute bacterial tonsillitis, bilateral peritonsillar abscesses are quite rare. The incidence of unsuspected contralateral peritonsillar abscess identified at tonsillectomy has been reported to be between 1.9% and 24%, while the overall incidence of bilateral peritonsillar abscess is reported to reach 4.9%. Diagnosis can be based on clinical criteria or imaging techniques. As far as the treatment is concerned, it is generally accepted that the basic strategy consists of systemic antibiotics and drainage of the pus. We report the case of a 19-year-old girl, treated in the emergency room with a bilateral diagnostic needle aspiration followed by bilateral incision and drainage along with intravenous clindamycin plus anti-inflammatory agents and hydration. Following treatment, the patient progressively experienced a marked alleviation of her odynophagia. She was discharged 48 hours later on a 10-day course of clindamycin.

## 1. Introduction

**Figure 1:**
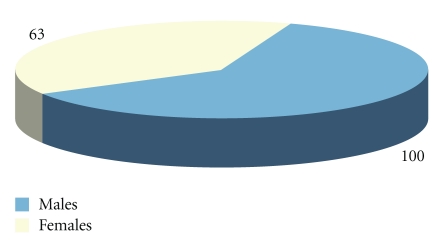
Diagnosed peritonsillar abscesses: Male to Female ratio in our 10-year series (1999–2009).

**Figure 2:**
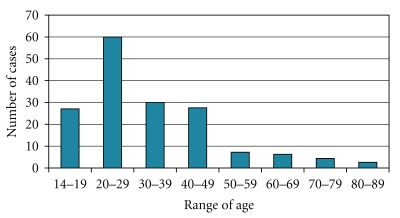
Diagnosed peritonsillar abscesses: Range of age in our 10-year series (1999–2009).

Peritonsillar abscesses (PTAs) are collections of purulent material that usually develop outside the tonsillar capsule near the superior pole. They develop as the most frequent complication of acute tonsillitis, when the infection spreads from the crypts to the loose alveolar peritonsillar tissues. They are mainly situated in the region of the upper pole and involve the soft palate pushing the tonsils forwards and towards the midline. The condition is usually unilateral and mostly affects young male adults (2 : 1 male preponderance) [1–6]. In our 10-year series (1999–2009), male to female ratio is about 100/63 (Figure 1), with the majority of the cases diagnosed between 20 and 40 years of age (Figure 2). 

Peritonsillar abscess is believed to be part of a clinical modality that progresses from acute tonsillitis to peritonsillar cellulitis and finally to peritonsillar abscess. The most common symptoms at presentation are sore throat, odynophagia (usually unilateral), dysphagia, otalgia, trismus, oral drooling, and high fever. Early diagnosis, with drainage of the abscess, is crucial to prevent perforation into the parapharyngeal/retropharyngeal space and further spread along the neck vessels to the mediastinum and skull base. Possible aspiration and severe upper airway obstruction due to epiglottis or laryngeal oedema may develop if treatment is delayed [3, 5–7]. 

The methods of accomplishing drainage have been varied, and the appropriate approach to the definitive treatment of peritonsillar abscess is still controversial. The theurapeutic options, in general, include needle aspiration, incision and drainage, quinsy tonsillectomy, intravenous antibiotics, and steroid therapy. Although unilateral peritonsillar abscess is a common complication of acute bacterial tonsillitis, bilateral peritonsillar abscesses are quite rare. The overall incidence of bilateral PTA is reported to reach 4.9% [3, 5–8]. In most bilateral cases, an unsuspected contralateral abscess is discovered during tonsillectomy. The incidence of unsuspected contralateral peritonsillar abscess identified at tonsillectomy has been reported to be between 1.9% and 24% [3, 5–8].

## 2. Case Presentation

**Figure 3:**
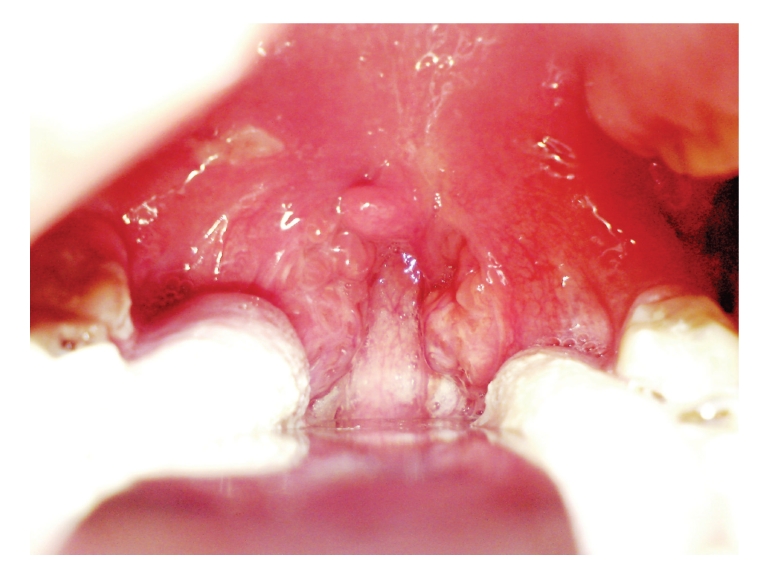
The bilateral Peritonsillar absesses in our case (12 hours after the initial incision and drainage which followed diagnostic needle aspiration). The incisions are still clearly visible.

A 19-year-old girl presented to the emergency department of our Hospital with a 6-day history of worsening odynophagia, bilateral earache, difficulty in swallowing solids, subjective fever, and significant trismus. Despite oral Clarithromycin prescribed by her GP, her symptoms worsened in the 24 hours preceding admission. Intraoral examination revealed a diffusely erythematous soft palate and uvula, with prominent swelling and midline protrusion (Figure 3). She showed no signs of upper airway obstruction in fiberoptic endoscopy. Laboratory tests revealed a significant increase of white blood cells, normal electrolytes, and no monocytosis. 

As the patient presented with significant symmetrical bulging of the soft palate, she was treated in the emergency room with a bilateral diagnostic needle aspiration (a 10 gauge needle was used) followed by bilateral incision and drainage (Figure 3). Computed tomography was not performed as the diagnosis was clear. 

Although bilateral peritonsillar abscesses are quite rarely diagnosed, it must be taken into account that tonsillitis is obviously a bilateral disease in most cases. Therefore, development of a peritonsillar cellulitis or abscess is quite likely to occur bilaterally [3] as happened in this case. The final progression of this—initially bilateral—disease on any side usually depends on the patient's physical condition and systemic response to infection, on former surgical interventions in the oropharynx and on prior medical treatments, especially with antibiotics [3, 5]. In our case, the patient was treated with Clarithromycin for 5 days before her admission and was also seriously dehydrated and malnutritioned for about one week, due to her significant odynophagia. These facts could have influenced the physical history of the disease, leading to this rare bilateral diagnostic modality. 

Foul-smelling pus was drained from both abscesses and sent for aerobic and anaerobic cultures and determination of sensitivities. The aerobic culture grew few alpha-hemolytic streptococci, occasional beta-hemolytic streptococci (not group A or B), rare *Staphylococcus aureus*, and rare *Candida albicans*. The anaerobic culture grew few beta-lactamase-positive *Prevotella melaninogenica* and few other beta-lactamase-positive, anaerobic, gram-negative rods. No penicillin-resistant organisms were isolated.

 The patient was admitted to our clinic and treated with intravenous clindamycin plus anti-inflammatory agents and intravenous hydration. Following treatment, the patient progressively experienced a marked alleviation of her odynophagia. She was discharged 48 hours later on a 10-day course of clindamycin. A followup intraoral examination one week later showed a well-resolved infection and normal mucosa.

## 3. Discussion

Unilateral peritonsillar abscess (PTA) is a common complication of acute bacterial tonsillitis and has been described since the time of Hippocrates [1–3]. However, bilateral peritonsillar abscesses are quite rare. Reports in the literature with regard to the incidence of bilateral abscesses are quite varied. The larger percentage includes cases where an unsuspected contralateral abscess was discovered during tonsillectomy. The incidence of unsuspected contralateral peritonsillar abscess identified at tonsillectomy has been reported to be between 1.9% and 24%, while the overall incidence of PTA is reported to reach 4.9% [3, 5–8]. 

As tonsillitis is an infection mostly involving both tonsils, it is probable that progression to peritonsillar abscess also occurs bilaterally, with the developmental stages of the abscesses being different on each side. However, most authors support that the individual's systemic response to infection, antibiotics, or surgical intervention may interrupt the progression of this disease at any stage [3]. Moreover, adequate antibiotic treatment as well as incision and drainage of the obvious abscess is often likely to suppress the development and even mask the presence of a peritonsillar abscess on the opposite side [3] Therefore, we do believe that the cases of bilateral abscesses are quite likely to be underreported.

The vast majority of peritonsillar abscesses harbor multiple organisms. The most common aerobic organisms isolated are *Streptococcus pyogenes*, *Streptococcus milleri*, *Haemophilus influenza*, and streptococci group viridans, while fusobacterium and *Prevotella melaninogenica* are reported to be the usually involved anaerobic organisms [3, 6]. In our case, the culture results demonstrated quite different organisms than expected, such as *Staphylococcus aureus* and *Candida albicans*. This fact could be attributed to the recent treatment with Clarithromycin, as well as to multiple antibiotic treatments involved during the last 6 months, as the patient's history revealed. 

The intraoral appearance of a bilateral peritonsillar abscess lacks the classic asymmetry and uvula deviation that are considered to be the hallmarks of a unilateral abscess. Moreover, antibiotic, anti-inflammatory, or steroid treatment may often mask the signs and symptoms of PTA [3–7]. Therefore, bilateral abscesses can be confused with other conditions such as bilateral lymphomas of the tonsils, infiltrating carcinomas of the soft palate or uvula, any tumors of the small salivary glands of the oral cavity, tonsillar cellulitis, or infectious mononucleosis [3–7]. That is the reason why some authors support that contrast-enhanced CT imaging can help diagnose bilateral PTA and should be considered in the presence of marked trismus but with the absence of unilateral inflammatory findings [9]. However, contrast CT scan is quite expensive and usually not available immediately. Therefore, we believe that the diagnosis of bilateral peritonsillar abscesses should be kept in mind when the clinical presentation suggests the diagnosis of PTA, but the physical examination reveals bilateral swollen tonsils with a midline uvula. In such cases, needle aspiration is a reliable alternative diagnostic procedure as it leads to immediate and accurate diagnosis in most situations. Some authors also support the diagnostic use of intraoral ultrasound in cooperative patients [10–12], reporting significantly good results. 

In our case, the patient presented with significant symmetrical bulging of the soft palate and significant trismus. She was treated in the emergency room with a bilateral diagnostic needle aspiration which was positive in both sides. This initial procedure was followed by bilateral incision and drainage. Computed tomography was not performed as the diagnosis was quite clear right after the bilateral diagnostic needle aspiration.

As far as the treatment of peritonsillar abscess is concerned, it is generally accepted that the basic strategy consists of systemic antibiotics covering group A *β*-hemolytic streptococci which is reported to be the most common offending organism and subsequent drainage of the pus [3–7]. Drainage of the pus from the abscess cavity can be accomplished via needle aspiration (sometimes ultrasound-guided), incision-drainage, or immediate (quinsy) tonsillectomy [3–8].

The surgical treatment of peritonsillar abscess that is not complicated with upper airway obstruction still remains controversial. Immediate tonsillectomy is an easy to perform one-stage surgical procedure assuring quick relief of trismus and pain and total evacuation of the pus. Such an operation may subsequently reveal an unsuspected contralateral peritonsillar abscess as well [3–8]. On the contrary, incision and drainage which is also supported by many authors is an awkward procedure, very unpleasant for the patient that could often lead to incomplete evacuation of the abscess cavity. That is the reason why the procedure is often necessary to be repeated several times. Besides, if an interval tonsillectomy is planned, such an operation could be technically more difficult because of the fibrosis of the tonsillar bed usually developed [3–8].

Quinsy tonsillectomy supporters also cite the need to prevent a recurrent abscess. However, those who favor only incision and drainage state that the reccurence rate has not been clearly defined. In fact, the reported frequency of recurrent peritonsillar abscess in several series is 5.9 to 22.7% [3]. 

Most authors accept that both needle aspiration and incision-drainage are the mainstay of management for the majority of peritonsillar abscesses and are reported to be equally effective for the treatment of the disease, whereas immediate tonsillectomy is now considered to be a reliable and safe procedure, suggested for bilateral cases, immunocompromised patients, or in cases with no response to systemic antibiotics or incision and drainage [3–8, 13]. 

On the other hand, initial conservative (nonsurgical treatment) is still supported by some authors in selected cases, before taking the risk of surgical drainage [13–15]. This strategy is reported to be involved especially in cases of inferior pole peritonsillar abscess, provided that the patient is immunocompetent and has no significant systemic diseases [14, 15]. The patient is treated with parenteral antibiotics and is under close observation for the first 48 hours. We suggest that poor response to antibiotics, progressively deteriorating clinical status or development of other complications, should always redirect the theurapeutic plan towards the surgical management. Moreover, the authors believe that this conservative strategy could also be involved for the first 48 hours in selected cases of peritonsillar celullitis, provided that diagnostic needle aspiration is negative in both sides and there is no evidence of immunodeficiency or any clinical signs or imaging data which would make the physician to suspect a life-threatening complication.

## 4. Conclusions

Although unilateral peritonsillar abscess is a common complication of acute bacterial tonsillitis, bilateral abscesses are quite rarely diagnosed. The intraoral appearance of a bilateral peritonsillar abscess lacks the classic asymmetry and uvula deviation. That is the reason why the ENT surgeon must keep this diagnostic modality in mind, even if the clinical appearance is not entirely suggestive, especially if previous antibiotic or steroid treatment has been involved. Diagnosis can be supported by both imaging techniques (such as CT or intraoral ultrasound) and bilateral needle aspiration. Treatment of choice consists of systemic antibiotics and drainage of the pus via bilateral incision and drainage or immediate quinsy tonsillectomy. Initial conservative (nonsurgical treatment) is supported by some authors in selected cases, before taking the risk of a surgical approach.

## References

[1] Richardson KA, Birck H (1981). Peritonsillar abscess in the pediatric population. *Otolaryngology—Head and Neck Surgery*.

[2] Herzon FS (1995). Harris P. Mosher Award thesis. Peritonsillar abscess: incidence, current management practices, and a proposal for treatment guidelines. *Laryngoscope*.

[3] Dalton RE, Abedi E, Sismanis A (1985). Bilateral peritonsillar abscesses and quinsy tonsillectomy. *Journal of the National Medical Association*.

[4] Kristensen S, Juul A, Nielsen F (1985). Quinsy: a bilateral presentation. *Journal of Laryngology and Otology*.

[5] Simons JP, Branstetter BF, Mandell DL (2006). Bilateral peritonsillar abscesses: case report and literature review. *American Journal of Otolaryngology*.

[6] Edinger JT, Hilal EY, Dastur KJ (2007). Bilateral peritonsillar abscesses: a challenging diagnosis. *Ear, Nose and Throat Journal*.

[7] Fasano CJ, Chudnofsky C, Vanderbeek P (2005). Bilateral peritonsillar abscesses: not your usual sore throat. *Journal of Emergency Medicine*.

[8] Kanesada K, Mogi G (1981). Bilateral peritonsillar abscesses. *Auris Nasus Larynx*.

[9] Patel KS, Ahmad S, O’Leary G, Michel M (1992). The role of computed tomography in the management of peritonsillar abscess. *Otolaryngology—Head and Neck Surgery*.

[10] Lyon M, Glisson P, Blaivas M (2003). Bilateral peritonsillar abscess diagnosed on the basis of intraoral sonography. *Journal of Ultrasound in Medicine*.

[11] Blaivas M, Theodoro D, Duggal S (2003). Ultrasound-guided drainage of peritonsillar abscess by the emergency physician. *American Journal of Emergency Medicine*.

[12] Scott PMJ, Loftus WK, Kew J, Ahuja A, Yue V, Van Hasselt CA (1999). Diagnosis of peritonsillar infections: a prospective study of ultrasound, computerized tomography and clinical diagnosis. *Journal of Laryngology and Otology*.

[13] Mehanna HM, Al-Bahnasawi L, White A (2002). National audit of the management of peritonsillar abscess. *Postgraduate Medical Journal*.

[14] Su W-Y, Hsu W-C, Wang C-P (2006). Inferior pole peritonsillar abscess successfully treated with non-surgical approach in four cases. *Tzu Chi Medical Journal*.

[15] Licameli GR, Grillone GA (1998). Inferior pole peritonsillar abscess. *Otolaryngology—Head and Neck Surgery*.

